# Genetic defects of the IRF1-mediated major histocompatibility complex class I antigen presentation pathway occur prevalently in the *JAK2* gene in non-small cell lung cancer

**DOI:** 10.18632/oncotarget.17689

**Published:** 2017-05-08

**Authors:** Tao Shen, Zhengming Chen, Zhizhuang Joe Zhao, Jie Wu

**Affiliations:** ^1^ Department of Molecular Oncology, H. Lee Moffitt Cancer Center and Research Institute, Tampa, Florida, USA; ^2^ Peggy and Charles Stephenson Cancer Center, University of Oklahoma Health Sciences Center, Oklahoma City, Oklahoma, USA; ^3^ Division of Biostatistics and Epidemiology, Department of Healthcare Policy and Research, Weill Cornell Medicine, New York, New York, USA; ^4^ Department of Pathology, University of Oklahoma Health Sciences Center, Oklahoma City, Oklahoma, USA

**Keywords:** NSCLC, JAK2, IRF1, Interferon-γ, antigen presentation

## Abstract

Recognition of major histocompatibility complex (MHC) class I antigens on tumor cells by cytotoxic T cells is involved in T cell-mediated tumor immune surveillance and immune checkpoint therapy. The interferon-γ (IFNγ)-IRF1 signaling pathway regulates MHC class I antigen presentation. To examine genetic defects of the IFNγ-IRF1 pathway in non-small cell lung cancer (NSCLC), we analyzed The Cancer Genome Atlas (TCGA) lung adenocarcinoma (LuAd) and lung squamous cell carcinoma (LuSc) data. Loss-of-function (LOF) genetic alterations of the IFNγ-IRF1 pathway genes (*IFNGR1, IFNGR2, JAK1, JAK2, STAT1, IRF1*) were found in 64 (6.3%) of 1,016 patients. These genetic defects occur prevalently in *JAK2* (33 cases) and often through deletions (29 cases) of chromosome 9p24.1. *JAK2* deletions were frequently, but not always, associated with deletions of PD-L1 gene (*CD274*), PD-L2 gene (*PDCD1LG2*), *PTPRD*, and *CDKN2A/CDKN2B* at the chromosome 9p24.1-9p21.3 region. *IRF1* expression was correlated with immune cytolytic activity markers *GZMA* and *PRF1* in NSCLC. IFNγ induced IRF1 expression and cell surface HLA-A/HLA-B/HLA-C (HLA-ABC) in A549, H661, H292, and H2172 cells that contained the wildtype JAK2, but not in H1573 and H1623 cells that were JAK2 defective. Deletion of *JAK2* or inhibition of the JAK2 kinase activity resulted in loss of IFNγ-induced IRF1 and cell surface HLA-ABC in JAK2 wildtype NSCLC cells, whereas expression of exogenous JAK2 in H1573 cells restored the IFNγ responses. These findings show that *JAK2* deficiency is the major mechanism of genetic defects of the IFNγ-IRF1 pathway in NSCLC and reveal a previously unrecognized significance of chromosome 9p deletion in NSCLC.

## INTRODUCTION

In T cell-mediated anti-tumor immunity, CD8^+^ cytotoxic T cells recognize MHC class I antigens on the tumor cell surface via T cell receptor to trigger the release of granzyme A (*GZMA*) and perforin (*PRF1*) to kill tumor cells. Thus, presentation of MHC class I antigens on the tumor cell surface plays a crucial role in T cell-mediated tumor immune surveillance and in immune checkpoint blockage therapy, which depends on the activated cytotoxic T cells to kill tumor cells [1–5]. MHC class I antigen presentation is regulated by IFNγ, which binds to its receptors (IFNGR1/IFNGR2) to activate JAK1/JAK2 and STAT1 and induces IRF1 [6]. IRF1 then controls expression of antigen presentation machinery (APM) proteins that include immune proteasomes low molecular weight protein-2 (LMP2)/LMP7/LMP10 and transporter associated with antigen processing-1 (TAP1)/TAP2. Immune proteasomes and TAP1/TAP2 produce and load MHC class I antigens onto MHC class I molecules for translocation to the cell surface [2]. Defects in this IFNγ-IRF1 regulated APM pathway allow tumors to escape immune surveillance [2], facilitate tumor development in animal models that include lung tumors [5, 7], and are associated with resistance to anti-PD-1 immunotherapy in melanoma [8, 9].

Anti-PD-1 and anti-PD-L1 immunotherapies have resulted in superior clinical responses in non-small cell lung cancer (NSCLC) [10–16]. However, responses are generally limited to ≤20% of NSCLC cases [14, 15, 17]. There is an urgent need to understand the susceptibility and resistance mechanisms in order to better predict responders/non-responders and to increase the response rate through combination treatments [3, 17–19]. Deficiency in the IFNγ-regulated MHC class I antigen presentation has been recognized as a plausible mechanism of resistance to immune checkpoint therapy [1, 20].

We previously found somatic loss-of-function (LOF) *JAK1* mutations in 9.5% of uterine cancer in Total Cancer Care (TCC^@^) tumors [21]. Majority of these J*AK1* mutations occurred as the result of frameshift mutations in polyhomonucleotide regions. In parallel, Kim and colleagues found 30% of endometrial cancer in TCGA were microsatellite instability-high (MSI-H) and 30% of TCGA MSI-H endometrial cancer cases had *JAK1* frame-shift truncating mutations [22]. This showed that 9% (30% x 30% = 9%) of endometrial cancer cases in TCGA had frame-shift truncating mutations. Moreover, missense LOF JAK1 mutations were reported in uterine leiomyosarcoma [23]. JAK1 truncating mutations impaired IFNγ-induced IRF1 and MHC class I antigen presentation in endometrial and ovarian cancer cells [21].

NSCLC and small cell lung cancer are two major forms of lung cancer [24]. Approximately 85% of lung cancer cases are NSCLC, which is comprised of adenocarcinoma (40%), squamous-cell carcinoma (25-40%), and large-cell carcinoma (10-15%) subtypes. Since NSCLC is a cancer type that anti-PD-1/anti-PD-L1 antibody therapies are effective and that the response/resistance mechanisms to immune checkpoint therapy remains incompletely understood, we focused our examination of the IFNγ-regulated MHC class I antigen presentation pathway in NSCLC in this study. We found that the genetic defects in the IFNγ receptor-IRF1 pathway genes [21] in NSCLC occurred predominantly via a mechanism distinct from that in endometrial cancer. Specifically, we identified *JAK2* deletion on chromosome 9p as the predominant mechanism of genetic defects in the IFNγ receptor-IRF1 pathway genes. Deletions of PD-L1 (*CD274*) and PD-L2 (*PDCD1LG2*) genes were always accompanied by *JAK2* deletion, suggesting that JAK2 deletion may be a mechanism to safeguard tumor cells from activated cytotoxic T cells in the absence of negative regulators PD-L1/PD-L2. Knocking out *JAK2* or inhibition of JAK2 kinase activity prevented presentation of MHC class I molecules on NSCLC cell surface. While chromosome 9p deletion was observed frequently in NSCLC in previous studies, its role has not been attributed to *JAK2*. Our analysis suggests that *JAK2* deletion offers tumor cells an advantage of evading immune surveillance and reveals a previously unknown functional significance of chromosome 9p deletion.

## RESULTS

### Genetic deficiencies of IFNγ-IRF1 signaling pathway genes in NSCLC occur prevalently in *JAK2*

**Figure 1:**
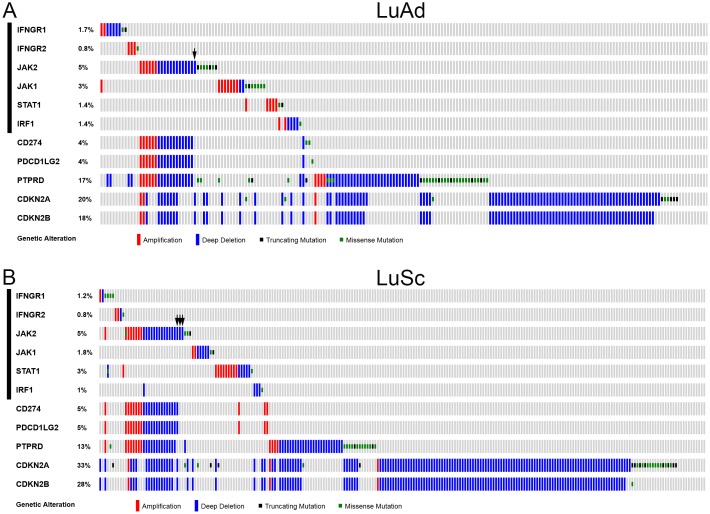
Genetic alterations of IFNγ-IRF1 pathway and selected chromosome 9p genes in NSCLC CNAs and mutations of the list genes were examined in the TCGA LuAd tumor samples (515 patients/517 samples RNA Seq V2 data) **(A)** and TCGA LuSc tumor samples (501 patients/501 samples RNA Seq V2 data) **(B)** through cBioPortal for Cancer Genomics (www.cbioportal.org) [27, 28]. Oncoprints of the genetic alternations in the list genes are shown. Arrows, non-continuous chromosome deletions involving *JAK2* or *JAK2* only deletion cases among the list genes. Black bars on the left indicate six IFNγ pathway genes examined in this study.

In T cell-mediated tumor immune surveillance, recognition of MHC class I antigens on the tumor cells by the T cell receptor of CD8^+^ cytotoxic T cells is mandatory for the effector T cells to kill tumor cells [2]. The IRF1-mediated IFNγ signaling pathway regulates MHC class I antigen presentation in tumor cells. Thus, a deficiency in the IFNγ-IRF1 signaling pathway would impair the T cell-mediated cytolytic activity. To assess the spectrum and prevalence of genetic defects in the IFNγ-IRF1 signaling pathway in NSCLC, we examined TCGA lung adenocarcinoma (LuAd) and lung squamous cell carcinoma (LuSc) data [25–28] for apparent loss-of-function (LOF) gene alterations (homozygous deletion or truncating mutation) of the IFNγ-IRF1 signaling pathway genes. These included *IFNGR1*, *IFNGR2*, *JAK2*, *JAK1*, *STAT1*, and *IRF1*. Among 515 cases of LuAd, 30 cases (5.8%) had a LOF alterations in one of the above 6 genes (Figure 1A). These 30 cases were mutually exclusive. LOF alterations of the *JAK2* gene occurred most often with 16 cases (2.5%), which included 13 homozygous deletion cases and 3 truncating mutation cases. Similarly, 34 of 501 LuSc cases (7.0%) had LOF alterations in one of the IFNγ signaling pathway genes (Figure 1B). LOF alterations in 33 of these 34 cases were mutually exclusive. JAK2 LOF occurred most frequently in 17 cases (3.4%). Thus, genetic defects in the IFNγ-IRF1 signaling pathway in NSCLC occur prevalently in the *JAK2* gene.

### Co-occurrence of *JAK2* deletion with other chromosome 9p genes

Among the 33 cases of *JAK2* LOF alterations, 29 cases (88%) were chromosomal deletion. Thus, unlike our previous finding in endometrial cancer where *JAK1* frameshift was the predominant mechanism of the IFNγ-IRF1 pathway genetic defects [21], *JAK2* gene deletion was the predominant mechanism of the IFNγ-IRF1 pathway genetic defects in NSCLC. Interestingly, 39 of 42 *JAK2* copy number alternation (CNA) cases, including both amplification and deletion, coincided with CNAs of *CD274* and *PDCD1LG2* that encode PD-L1 and PD-L2, respectively, in LuAd and LuSc (Figure 1). *JAK2* was deleted in all of *CD274* and *PDCD1LG2* deletion cases. Examination of chromosomal locations of *JAK2*, *CD274*, and *PDCD1LG2* genes indicated that these genes are co-localized at human chromosome 9p24.1 (Supplementary Figure 1).

**Figure 2:**
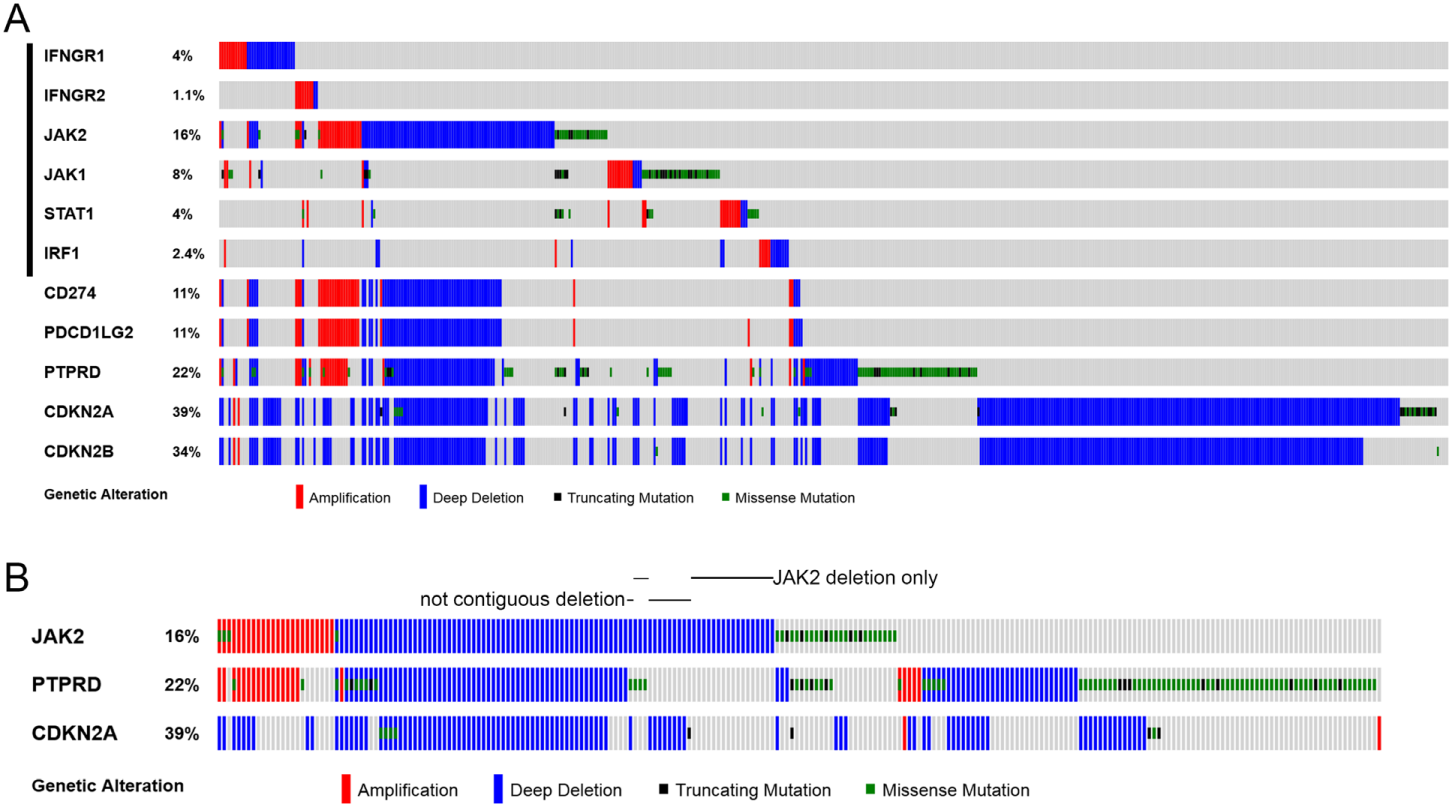
Genetic alterations of IFNγ-IRF1 pathway and selected chromosome 9p genes in cancer cell lines **(A)** CNAs and mutations of the listed 11 genes were examined in 881 cancer cell lines in Cancer Cell Line Encyclopedia (CCLE) [37] through cBioPortal. Oncoprints of the genetic alterations are shown. **(B)** Oncoprints *JAK2, PTPRD*, and *CDKN2A* genetic alterations in CCLE cells are shown. Cell lines with non-continuous chromosome 9p deletions or have *JAK2* deletion in the absence of *PTPRD* and *CDKN2A* deletion are indicated.

Chromosome 9p21 and 9p23-9p24 were identified previously as frequently deleted regions in NSCLC genomes [29–35]. Tumor suppressor genes *CDKN2A/CDKN2B* and *PTPRD* are located at chromosome 9p21.3 and 9p23-9p24.1, respectively (Figure 2). Loss of *CDKN2A/CDKN2B* or *PTPRD* tumor suppressor was often used to explain the frequent chromosome 9p deletion in human cancer [29, 30, 33, 34, 36]. The TCGA data showed that *JAK2* deletion in NSCLC was often associated with deletions of either *PTPRD* and/or *CDKN2A/CDKN2B* genes (Figure 1). Thus, the mechanism of frequent *JAK2* deletion in NSCLC may be a bystander event due to deletions of the tumor suppressor genes *PTPRD* and *CDKN2A*/*CDKN2B* because the proximity of these genes co-located at the chromosome 9p21.3-9p24.1 region.

However, it was observed previously in NSCLC that deletions in 9p24, 9p23, and 9p21 were not contiguous [29]. Similarly, chromosomal deletions of *JAK2*, *PTPRD*, and *CDKN2A/CDKN2B* genes did not always occur in a contiguous manner in TCGA NSCLC tumors (Figure 1, see cases indicated by black arrows). *JAK2* and *CDKN2A/CDKN2B* loci were deleted while *PTPRD* was not changed in the TCGA-50-930 and TCGA-22-1002 tumors. In TCGA-21-1082 and TCGA-77-7335 tumors, only the *JAK2* locus was deleted.

Examination of TCGA data of 881 cancer cell lines in the cancer cell line encyclopedia (CCLE) cohorts [37] again showed that the IFNγ-IRF1 pathway genetic defects in these cancer cells occurred most often through JAK2 deletion (Figure 2). *CD274* and *PDCD1LG2* deletions were always accompanied by *JAK2* deletion (Figure 2A). *JAK2* deletion was often, but not always, associated with *PTPRD* and/or *CDKN2A/CDKN2B* deletions (Figure 2B). Among the 90 CCLE cancer cell lines with *JAK2* homozygous deletion, 17 were lung cancer cell lines.

The non-contiguity of *CDKN2A/CDKN2B*-*PTPRD*-*JAK2* deletion on chromosome 9p and the fact that *JAK2* deletion occurred in the absence of *CDKN2A/CDKN2B* or *PTPRD* deletion suggest that *JAK2* deletion is not merely attributed to deletion of *CDKN2A/CDKN2B* or *PTPRD* loci that accidently extended beyond these tumor suppressor genes. Rather, it suggests that *JAK2* deficiency by itself offers an advantage to certain cancer cells.

### IRF1 expression correlates with granzyme A and perforin expression in NSCLC

After binding to MHC class I antigens on tumor cells via T cell receptor, activated CD8^+^ cytotoxic T cells release granzyme A (GZMA) and perforin (PRF1) to destroy tumor cells. mRNA levels of these two cytolytic effectors are markers of immune cytolytic activity of CD8^+^ cytolytic T cells and clinical responsiveness to immune checkpoint therapies [3, 19, 38]. We used *IRF1* mRNA as a marker of IFNγ-regulated MHC class I antigen presentation pathway in tumor cells, CD8 as activated cytotoxic T cell marker, and GZMA and PRF1 as immune cytolytic activity markers to analyze if the IFNγ signaling pathway activity is correlated with cytotoxic T cell-associated cytolytic activity.

**Figure 3:**
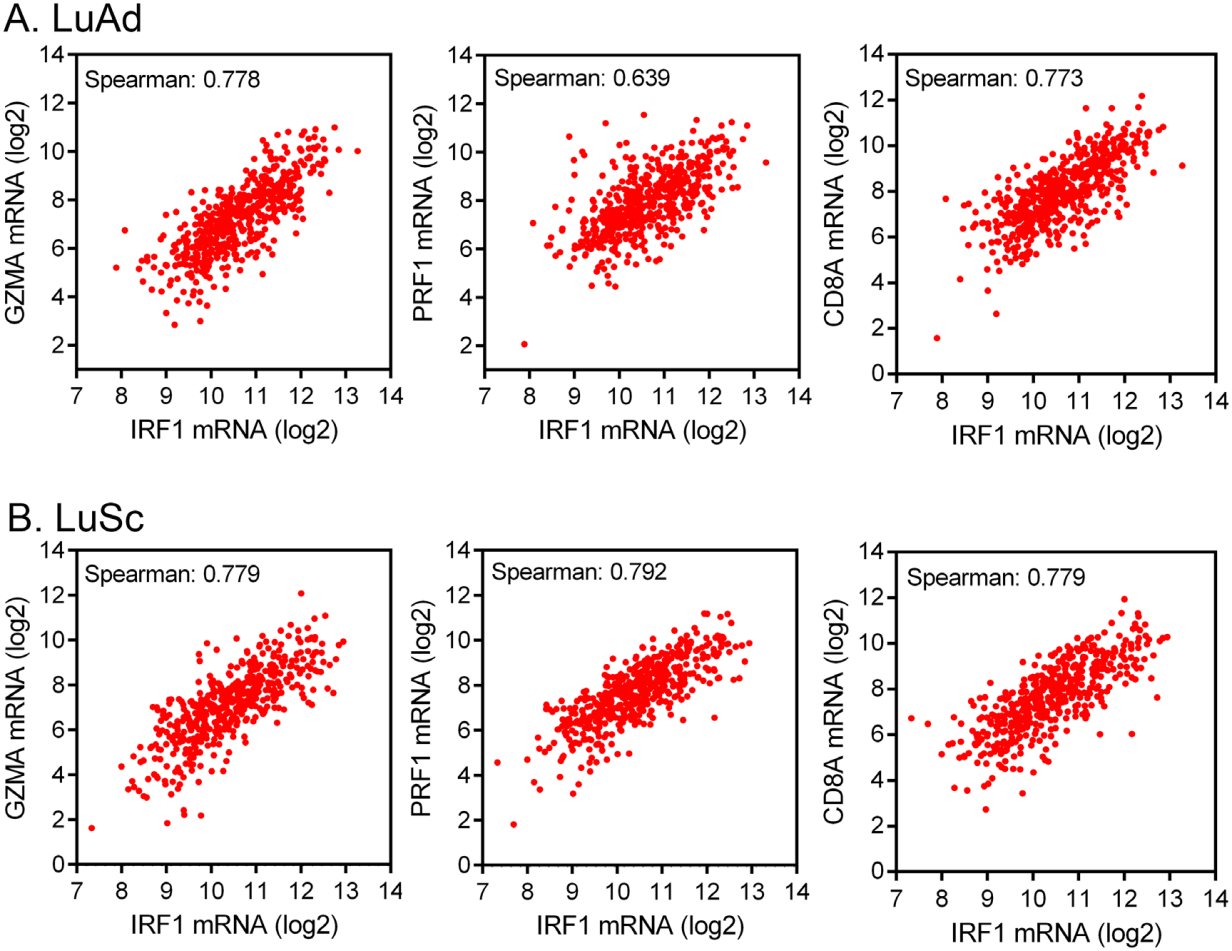
Correlation of *IRF1* expression with immune cytolytic activity markers **(A)** Co-expression of *IRF1* mRNA and immune cytolytic activity markers [3] granzyme A (*GZMA*) and perforin (*PRF1*) and cytotoxic T cell marker CD8 in 515 TCGA cases of LuAd. **(B)** Co-expression of IRF1 with granzyme A, perforin, and cytotoxic T cell markers in 501 TCGA cases of LuSc. Data were obtained via cBioPortal.

Analyses of TCGA RNA-seq data from 515 cases of LuAd and 501 cases of LuSc showed that the *IRF1* mRNA levels in these NSCLC cohorts were positively correlated with *GZMA*, *PRF1*, and CD8 mRNA levels (Figure 3). The Spearman correlation coefficients ranged from 0.639 to 0.792. Therefore, the *IRF1* level was well correlated with the *GZMA* and *PRF1* levels and *GZMA* and *PRF1* levels were mainly associated with activated T cells in these 1,016 cases of NSCLC.

### Inhibition of JAK2 prevents IFNγ-induced IRF1 expression and cell surface of HLA-ABC

**Figure 4:**
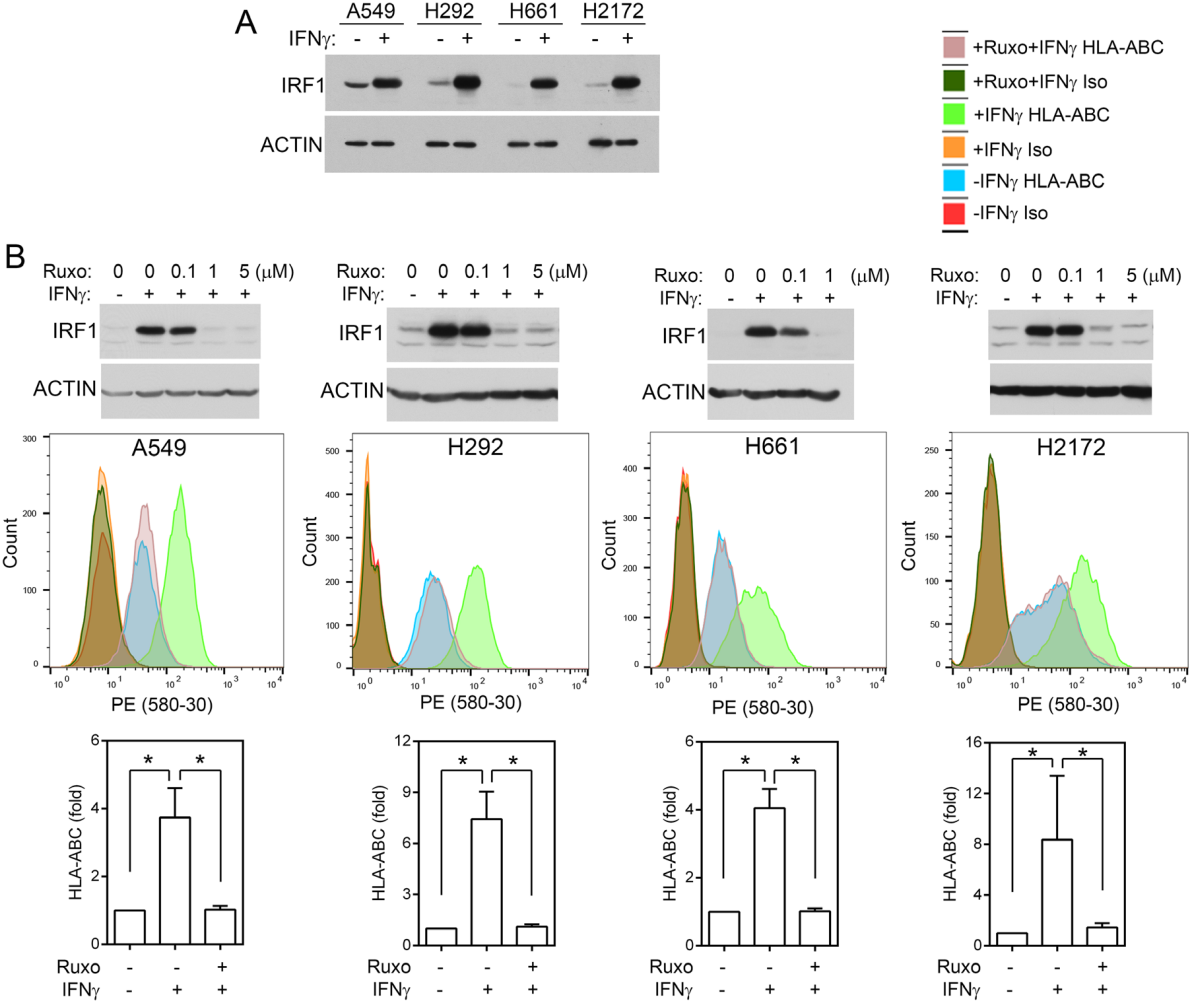
JAK2 protein tyrosine kinase activity is required for IFNγ-induced IRF1 and cell surface MHC class I molecules **(A)** Cells were treated with IFNγ (20 ng/ml) for 6 h. An equal amount of cell lysates were analyzed by immunoblotting with antibodies to IRF1 and ACTIN. **(B)** Upper panels, cells were pretreated with indicated concentrations of ruxolitinib for 1h and then stimulated with IFNγ for 6h. Cell lysates were analyzed by immunoblotting as in (A). Lower panels, cells were pre-treated with ruxolitinib for 1 h and then mock-treated or treated with IFNγ (100 ng/ml) for 24 h and processed for immunostaining with a PE-conjugated anti-HLA-ABC antibody or an isotype control antibody followed by flow cytometric analysis. Examples of spectra from representative experiments are shown. Graphs: fold changes between cells with and without IFNγ stimulation. n = 6 for all conditions in all cell lines performed in three or more separate experiments. *, *p* < 0.05 (Wilcoxon signed-rank test).

A549, H661, H292, and H2172 are NSCLC cell lines without homozygous *JAK2* deletions or mutation based on CCLE data. HLA-A, -B, and -C (HLA-ABC) are human MHC class I molecules. MHC class I antigens are normally loaded into HLA-ABC/β-2 microglobulin (*B2M*) and transported to cell surface for presentation to cytotoxic T cells. When HLA-ABC/β-2 microglobulin are not loaded with antigens, they become unstable and are degraded more rapidly in the cells, resulting in a lower level of HLA-ABC molecules on the cell surface [21, 39, 40]. IFNγ stimulated IRF1 expression and increased cell surface HLA-ABC in these cells (Figure 4), indicative of MHC class I antigen presentation [21]. To determine if the JAK2 protein tyrosine kinase activity is required for IFNγ-stimulated IRF1 expression and MHC class I antigen presentation, we treated A549, H661, H292, and H2172 NSCLC cells with the JAK2 inhibitor ruxolitinib. Complete inhibition of IFNγ-induced IRF1 was observed at 1 μM ruxolitinib (Figure 4B). Consistently, while IFNγ increased the cell surface HLA-ABC level in all four cell lines significantly (*p* < 0.05), pretreatment with ruxolitinib (1 μM) blocked the IFNγ-induced cell surface HLA-ABC, indicating that the JAK2 protein kinase activity is necessary for these IFNγ-induced responses.

### JAK2 deficiency impairs IFNγ-stimulated IRF1 expression and MHC class I antigen presentation on NSCLC cells

**Figure 5:**
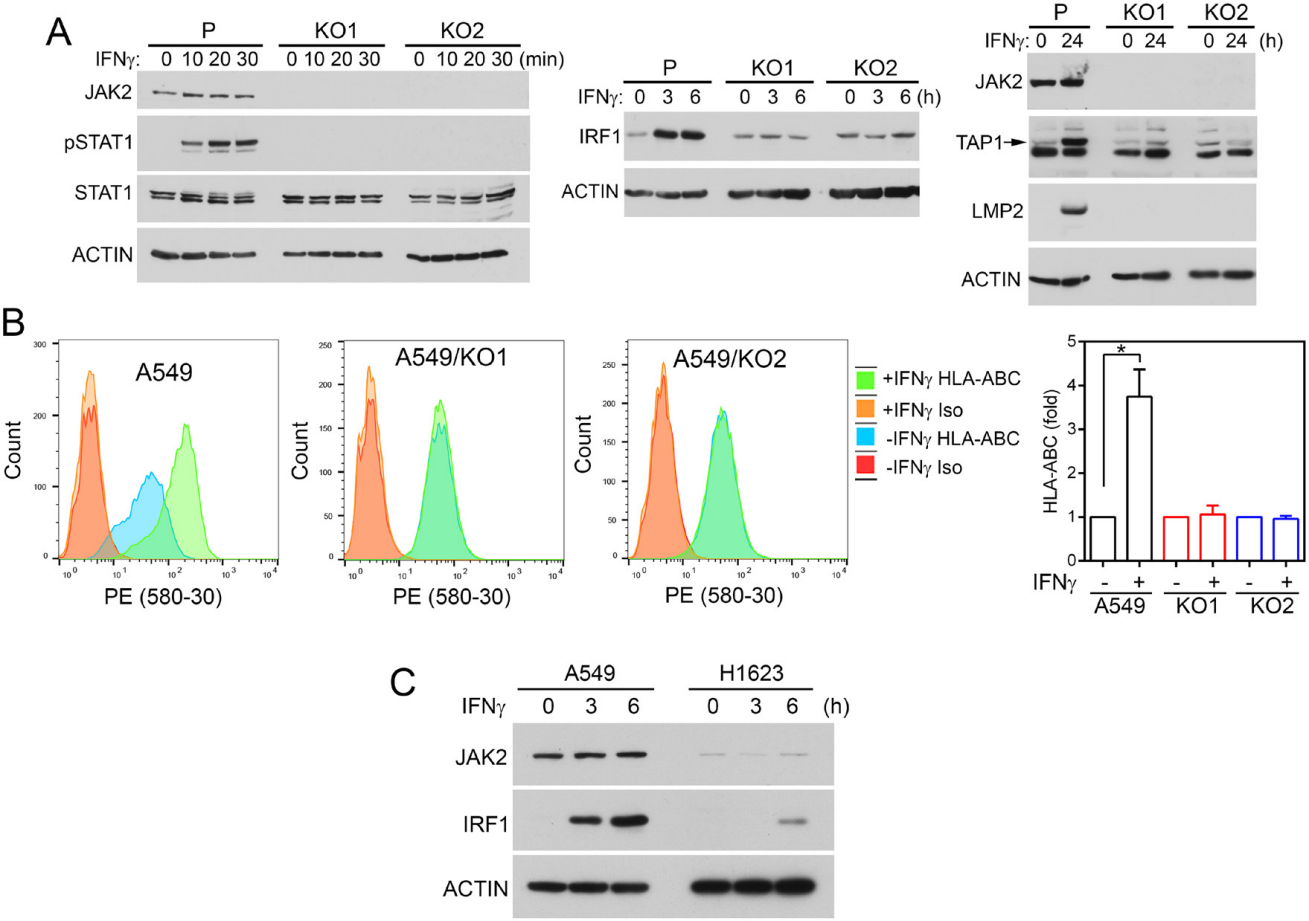
JAK2 deficiency impairs responses to IFNγ in NSCLC cells **(A, B)** JAK2 was knocked out using CRISPR/Cas9 as described in Materials and Methods. Two JAK2 knockout cell lines (KO1, KO2) were isolated and compared with the parental cells (P). (A) Cells were treated with IFNγ as indicated. Equal amounts of cell lysate proteins were analyzed by immunoblotting with the indicated antibodies. (B) Cells were treated with or without IFNγ (100 ng/ml) for 24 h. The amounts of HLA-ABC on the cell surface were analyzed by flow cytometry following immunostaining with PE-conjugated anti-HLA-ABC or isotype control antibodies. n = 6 for all conditions in all three cell lines performed in two or more separate experiments. *, *p* < 0.05 between cells treated with or without IFNγ, Wilcoxon signed-rank test. **(C)** A549 and H1623 cells were stimulated with IFNγ and cell lysates were analyzed by immunoblotting as indicated.

To determine if loss of JAK2 impairs the IFNγ-induced MHC class I antigen presentation pathway, we knockout *JAK2* in A549 cells using lentiviral CRISPR/Cas9 sgRNAs. Two stable cell lines (A549/KO1, A549/KO2) that did not have detectable JAK2 protein were isolated (Figure 5A). While IFNγ activated STAT1 and induced IRF1, TAP1, and LMP2 expression in A549 cells, it could not activate STAT1 or induce IRF1, TAP1, or LMP2 in the JAK2 knockout A549/KO1 and A549/KO2 cells (Figure 5A). Flow cytometric analysis of cell surface HLA-ABC expression indicated that IFNγ could not increase cell surface HLA-ABC in A549/KO1 and A549/KO2 cells (Figure 5B).

Next, H661 cells were infected with lentiviral *JAK2* CRISPR/Cas9 sgRNAs. The pool of puromycin-resistant cells (H661/KO) were used in subsequent analyses. As shown in Supplementary Figure 2, JAK2 was not detected in H661/KO cells. IFN-γ induced pSTAT1, IRF1, TAP1, and LMP2 in H661 but not in the JAK2 knockout H661/KO cells. Furthermore, IFNγ could not increase cell surface HLA-ABC in H661/KO cells (Supplementary Figure 2B).

Based on CCLE data (cbioportal.org), the NSCLC cell line H1623 has *JAK2* deep deletion. Compared with A549 cells, H1623 cells had a minimal amount of JAK2 protein (Figure 5C). While IRF1 was markedly increased in A549 cells after IFNγ stimulation, a very low level of IRF1 was detected in H1623 cells.

### Restoration of JAK2 in H1573 cells rescues IFN-γ signaling

**Figure 6:**
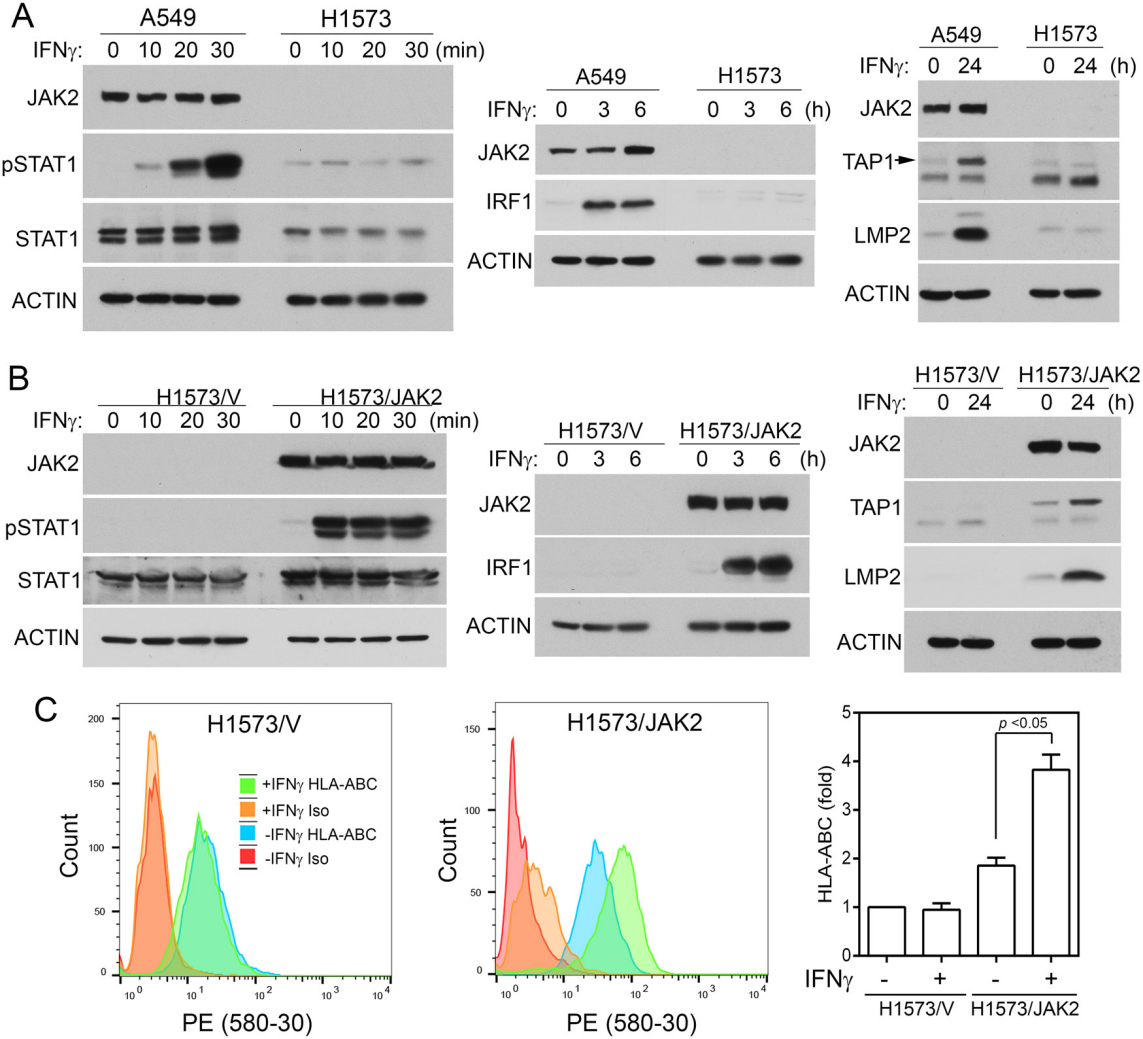
Expression of JAK2 in H1573 cells restores IFNγ responses **(A)** H1573 cells had no detectable JAK2 and were defective in IFNγ-induced IRF1 axis. A549 (positive control cells) and H1573 cells were treated with IFNγ as indicated. Cell lysates were analyzed by immunoblotting with indicated antibodies. **(B)** H1573/V and H1573/JAK2 cells were treated with IFNγ as shown and cell lysates were analyzed by immunoblotting with indicated antibodies. **(C)** H1573/V and H1573/JAK2 cells were treated with or without IFNγ for 24 h and cell surface HLA-ABC were analyzed by flow cytometry after immunostaining with HLA-ABC. Left, representative spectra from flow cytometric analysis. Right, fold changes. n = 6 for all conditions in both cells (*, p < 0.05, Wilcoxon signed-rank test).

H1573 human NSCLC cells harbor JAK2 S507* nonsense mutation that results in truncation of both pseudo kinase and tyrosine kinase domain of JAK2. IFNγ could not increase STAT1 tyrosine phosphorylation or increase IRF1, TAP1, or LMP2 level in H1573, while parallel experiments in the JAK2 wildtype A549 cells showed readily detectable IFNγ-induced responses. (Figure 6A), indicating that H1573 cells are defective in IFNγ signaling. We established a H1573/JAK2 cell line by infecting H1573 cells with retrovirus-encoded JAK2. As shown in Figure 6B, the control H1573/V cells did not have detectable JAK2 protein and were unresponsive to IFNγ-stimulation in STAT1 tyrosine phosphorylation and expression of IRF1, TAP1, and LMP2. In the JAK2-restored H1573/JAK2 cells, IFNγ was able to induce pSTAT1, IRF1, TAP1, and LMP2. Moreover, IFNγ could not increase the cell surface HLA-ABC level in H1573/V cells, whereas treatment of H1573/JAK2 cells with IFNγ significantly increased the cell surface HLA-ABC (Figure 6C, *p* < 0.05). These results show that JAK2 deficiency is responsible for the defect in the IFNγ-regulated MHC class I antigen presentation in H1573 cells.

## DISCUSSION

We found here that genetic defects in the IFNγ-IRF1 signaling pathway genes occurred in 5.8% of LuAd and 7.0% of LuSc among 1016 NSCLC cases in TCGA. Approximately 52% of these defects occurred in *JAK2*, of which chromosome deletion of *JAK2* accounting for 88% of the cases. *JAK2* resides on chromosome 9p24.1. Interestingly, *CD274, PDCD1LG2, PTPRD*, and *CDKN2A/CDKN2B* are also located in the nearby chromosome 9p region (Supplementary Figure 1). Chromosome 9p deletions have been observed frequently in NSCLC [25, 26, 29-31, 33] and in some other types of tumors such as glioma [34, 41]. *CDKN2A/CDKN2B* at chromosome 9p21.3 and *PTPRD* at chromosome 9p23-p921.1 were identified as tumor suppressor genes in the chromosome 9p arm to give the functional explanation of chromosome 9p deletions [25, 26, 29, 30, 32-34, 41]. The high incidence of *JAK2* deletion in NSCLC was likely due in part to the frequent deletion of tumor suppressors *CDKN2A/CDKN2B* and *PTPRD* in the chromosome 9p arm in NSCLC.

However, it appears unlikely that *JAK2* deletion was merely a bystander effect in which chromosome 9p deletion accidently extended beyond the *CDKN2A/CDKN2B* and *PTPRD* gene regions. First, as previously reported [29], deletions of chromosome 9p were not contiguous (Figures 1, 2), suggesting that deletion of *JAK2* conferred a functional selection advantage to the cancer cells. Second, there were cases in TCGA tumors and CCLC cancer cell lines in which *JAK2* was deleted in the absence of *CDKN2A/CDKN2B* or *PTPRD* deletion (Figures 1, 2), suggesting that *JAK2* deletion alone may have a functional benefit to cancer cells. Third, *CD274* and *PDCD1LG2* were deleted in some cases of TCGA NSCLC and CCLC cancer cell lines. *CD274* and *PDCD1LG2* deletions were always accompanied by *JAK2* deletion. Since PD-L1/PD-L2 suppress T cell-based immunity against tumor cells, deletions of PD-L1/PD-L2 could result in constitutively active tumor-targeting cytotoxic T cells. In fact, blocking PD-1/PD-L1 interaction is the principle of immune checkpoint blockage therapies using anti-PD-1/anti-PD-L1 antibodies. Loss of PD-L1/PD-L2 T cell suppressors while maintaining the IFNγ-regulated MHC class I antigen presentation pathway that allows cytotoxic T cells to recognize tumor cells would be disadvantageous to the tumor cells. Thus, JAK2 deletion is a potential mechanism to hide tumor cells from recognition by cytotoxic T cells, especially in the cases of *CDC274* and *PDCD1LG2* deletions.

While anti-PD-1/anti-PD-L1 antibodies therapies have shown remarkable efficacies in a subset of NSCLC patients, the majority of NSCLC are unresponsive to these immune checkpoint blockage therapies [12, 14, 15]. Various therapy resistance mechanisms may exist. In the absence of PD-L1, the anti-PD-L1 antibody atezolizumab would lack the target on tumor cells while anti-PD-1 antibodies nivolumab and pembrolizumab would lack one of bases of their action. More importantly, recognition of MHC class I antigens by T cell receptor of CD8^+^ cytotoxic T cells is required for the effector T cells to destroy tumor cells. The well-correlated mRNA levels of *IRF1* with that of immune cytotoxic markers *GZMA/PRF1* presented in this study support the important role of the IFNγ-IRF1 signaling axis in modulation of the T cell-based immune cytotoxic activities. Consistent with this notion, NSCLC patients with pre-existing high T-effector/IFNγ-associated gene expression had improved overall survival in a recent atezolizumab clinical trial [16].

Using knockout and a chemical inhibitor, our data show that JAK2 was necessary for IFNγ-induced IRF1 and cancer cell surface expression of HLA-ABC MHC class I molecules. On the other hand, expression of exogenous JAK2 in a cell line lacking endogenous JAK2 was sufficient to restore IFNγ-induced IRF1 and cell surface expression of HLA-ABC. These results demonstrate that JAK2 deficiency would impair IFNγ-regulated MHC class I antigen presentation. We thereby postulate that *JAK2* genetic defects (deletion and truncating mutations) in NSCLC represent a likely mechanism underlying the resistance of cancer cells to anti-PD-1/anti-PD-L1 immune checkpoint therapy.

Gain-of-function JAK2 mutant JAK2^V617F^ is an established oncoprotein associated with myeloproliferative neoplasms (MPNs) polycythemia vera, essential thrombocythemia, and primary myelofibrosis [42]. Moreover, JAK2 activated STAT3 pathway has tumor promoting activity [43–45]. JAK1/JAK2 inhibitor such as ruxolitinib is being used to treat MPNs and other JAK2 inhibitors are in various stages of development [46]. Unorthodoxically, this study in NSCLC and the previous studies of JAK1 in uterine cancer [21–23] also suggest that JAK1 and JAK2 have tumor suppressor function, deficiencies of them could allow tumor cells to escape T-cell mediated immune surveillance. Caution should be taken when considering JAK inhibitors for combination treatments involving tumor immunity.

## MATERIALS AND METHODS

### Reagents

Human IFNγ was from Peprotech. Antibodies to STAT1 (SC79877), IRF1 (SC497), and LMP2 (SC271354) were from Santa Cruz Biotechnologies. STAT1-pTyr701 (pSTAT1) antibodies were from Santa Cruz (SC7988) or Cell Signaling Technologies (#7649). Antibodies to JAK2 (#3230) was from Cell Signaling Technologies. Anti-TAP1 antibody (ADI-CSA-620-E) was from ENZO. PE-conjugated mouse anti-human HLA-ABC antibody (clone G46-2.6) and the isotype control antibody were from BD Pharmingen. LentiCRISPRv2 [47] was obtained from Addgene. Ruxolitinib was from LC laboratories.

### Genomic data analysis

CNAs, mutations, and mRNA expression of lung adenocarcinoma [tumor samples with mRNA data (RNA seq V2), 515 patients, 517 tumor samples] and lung squamous cell carcinoma [tumor samples with mRNA data (RNA Seq V2), 501 patients, 501 tumor samples] were from TCGA [25, 26] and was down loaded via www.cBioPortal.org [27, 28]. CNA and mutation of Cancer Cell line encyclopedia [37] cell lines (881 cell lines with sequencing and CNA data) were based on cBioPortal. Oncoprint visualization of CNAs and mutations were adapted from cBioPortal. Human chromosome 9p information and gene locations were obtained from Ensembl (www.ensembl.org) [48] and was based on human genome assembly GRCh38.

### Cell lines, cell cultures, and immunoblotting

Human NSCLC cell lines A549, H661, H292, and H2172 were from a central repository at the H. Lee Moffitt Cancer Center and Research Institute and had been authenticated by STR analysis (ACTG Inc, Wheling, IL) as of September 2010 [49]. Cells in the central repository had been routinely tested and were negative for mycoplasma (PlasmoTest, InvivoGen, San Diego, CA). H1593 cells were obtained from American Type Culture Collection (ATCC) in 2015. H1623 cells were obtained from ATCC in 2017. A549 cells were cultured in DMEM/10% fetal bovine serum (FBS). H611, H292, H2172, H1573, and H1623 cells were cultured in RPMI-1640/10% FBS. Cell lysate preparation and immunoblotting were performed as described previously [21, 49, 50].

### Flow cytometry

Cells were treated with or without IFNγ (100 ng/ml) for 24 h, detached from plates using Accutase, and suspended in PBS containing 0.5% BSA. Cells were stained with a PE-conjugated mouse anti-human HLA-ABC antibody (clone G46-2.6) or the isotype control antibody according to the supplier’s instruction as described previously [21]. Stained cells were fixed with 4% paraformaldehyde. Data were acquired on a FACSCanto II flow cytometer at the Moffitt Cancer Center or a Stratedigm S1000 flow cytometer at University of Oklahoma Health Sciences Center. Statistical analysis was performed using Wilcoxon signed-rank test. *p* < 0.05 was considered significantly different.

### CRISPR/Cas9-mediated JAK2 knockout

Three human JAK2 targeting single guide RNAs (sgRNAs) were selected based on the Zhang Lab Cas9 target design tool (http://crispr.mit.edu:8079/) [51]. The target sites were located on JAK2 exon 7. The targeting sequences were: G1: TTTTGACAAGGAAGCGAATA; G2: ATTATAATAACTGGAAACGG; G3: TCACCTGAAGGACCACTTCC. 5’-phosphorylated oligonucleotides were annealed and cloned into BsmBI digested lentiCRISPRv2 by standard procedure to generate lentiCRISPRv2-JAK2sgRNA-1, -2, and -3 plasmids. Lentiviruses containing JAK2 sgRNAs CRISPR/Cas9 were prepared by transfection of 293T cells with a mixture of equal amounts of the lentiCRISPRv2-JAK2sgRNA-1, -2, and -3 plasmids plus PMD2 and PSPAX. A549 cells were infected with the JAK2 sgRNAs CRISPR/Cas lentiviruses. Infected cells were selected by puromycin. Puromycin-resistant cell lines were isolated and screened for the absence of JAK2 protein by immunoblotting. H661 cells were similarly infected with the lentiviruses but puromycin-resistant pool of cells were used in subsequent analysis without clone selection since JAK2 was not detectable in the pool of cells.

### Expression of exogenous JAK2

*JAK2* coding sequence was cloned into a retroviral vector containing puromycin-resistant gene. Retroviruses were produced by transfection of empty or JAK2-encoding retroviral plasmids into Ampho-293 cells. H1573 cells were infected with retroviruses containing the JAK2 coding sequence. Approximately 80 puromycin-resistant cell lines (clones) were screened for the expression of JAK2 protein by immunoblotting and a cell line with detectable JAK2 (H1573/JAK2) was isolated. Control H1573 cells (H1573/V) were made by infected with retroviruses without the JAK2 cDNA insert and selected with puromycin (1 μg/ml).

## SUPPLEMENTARY MATERIALS FIGURES


