# Supplementary material for: Genetic defects of the IRF1-mediated major histocompatibility complex class I antigen presentation pathway occur prevalently in the JAK2 gene in non-small cell lung cancer
Source: Oncotarget. 2017 May 8;8(37):60975–86. doi: 10.18632/oncotarget.17689 (PMC5617399; doi:10.18632/oncotarget.17689)
Supplement: Supplementary file 1 [file oncotarget-08-60975-s001.pdf]

Genetic defects of the IRF1-mediated major histocompatibility complex class I antigen presentation pathway occur prevalently in the *JAK2* gene in non-small cell lung cancer

SUPPLEMENTARY MATERIALS

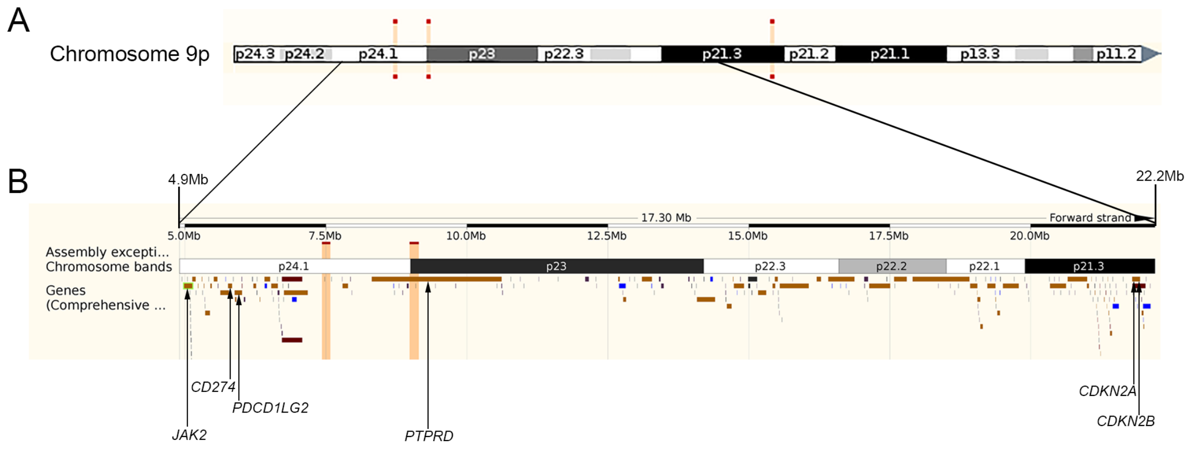

Supplementary Figure 1: Human chromosome 9p region. **(A)** Banding of human chromosome 9p. **(B)** Genes located at human chromosome 9p 4.9Mb-22.2Mb region. Data were obtained from Ensembl (www.ensemble.org) that based on human genome assembly GRCh38. Locations of *JAK2, CD274, PDCD1LG2, PTPRD, and CDKN2A/CDKN2B* genes are indicated.

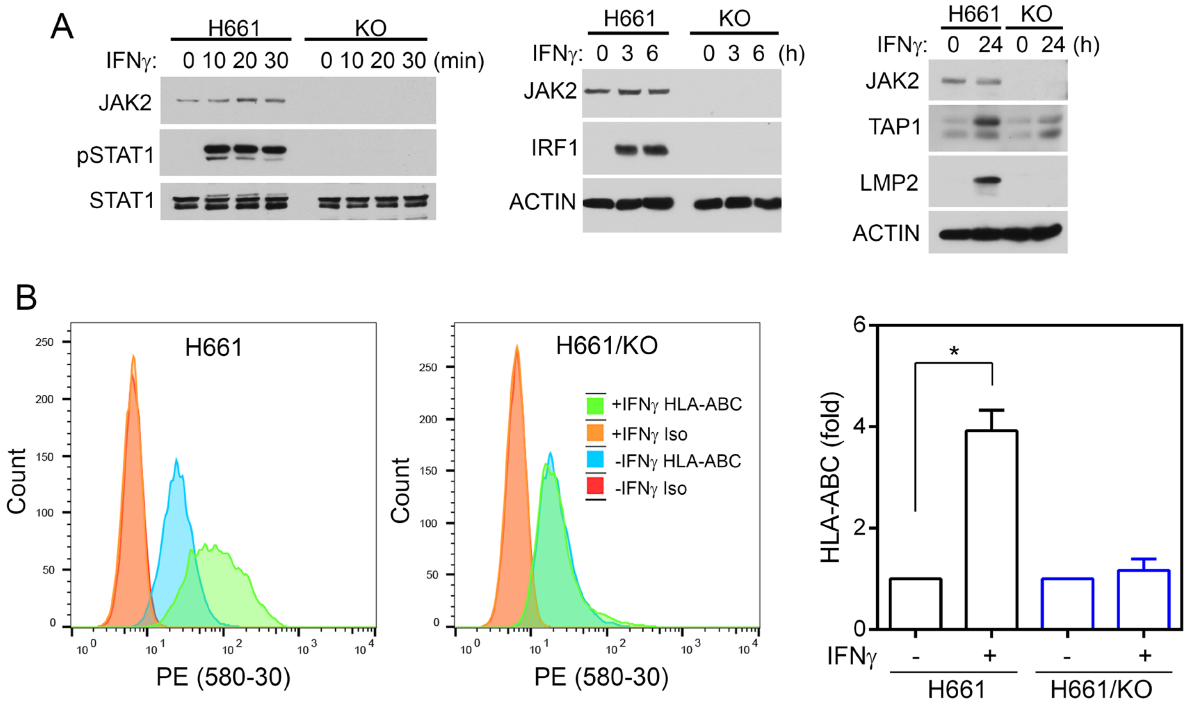

Supplementary Figure 2: *JAK2* knockout prevents IFNγ-induced MHC class I antigen presentation in H661 cells. *JAK2* was knocked out using CRISPR/Cas9 as described in Materials and Methods. JAK2 knockout H661 cells (H661/KO) and the parental cells were compared for IFNγ-induced pSTAT1, IRF1, TAP1, and LMP2 **(A)** and cell surface HLA-ABC **(B)** similar to that described for A549 cells in Figure 5 (*, $p < 0.05$, Wilcoxon signed-rank test).
